# Pulmonary pneumocystosis in a captive kinkajou with molecular evidence of a novel *Pneumocystis* lineage

**DOI:** 10.1177/10406387251413289

**Published:** 2026-01-22

**Authors:** Pablo Díaz-Santana, Alejandro Suárez-Bonnet, Javier Déniz-Marrero, Francisco J. Salguero, Bernat Martí-Garcia, Vincente Friaza, Enrique J. Calderón, Sai Fingerhood

**Affiliations:** Veterinary Pathology Centre, School of Veterinary Medicine, Faculty of Health and Medical Sciences, University of Surrey, Guildford, Surrey, UK; Department of Pathobiology and Population Sciences, The Royal Veterinary College, Hatfield, UK;; Veterinary Pathology Centre, School of Veterinary Medicine, Faculty of Health and Medical Sciences, University of Surrey, Guildford, Surrey, UK; Veterinary Pathology Centre, School of Veterinary Medicine, Faculty of Health and Medical Sciences, University of Surrey, Guildford, Surrey, UK; United Kingdom Health Security Agency (UKHSA-Porton), Porton Down, Salisbury, Wiltshire, UK; Department of Pathobiology and Population Sciences, The Royal Veterinary College, Hatfield, UK; Biomedicine Institute of Seville, Hospital Universitario Virgen del Rocío, Universidad de Sevilla, Seville, Spain; Centro de Investigación Biomédica en Red de Epidemiología y Salud Pública, Madrid, Spain; Biomedicine Institute of Seville, Hospital Universitario Virgen del Rocío, Universidad de Sevilla, Seville, Spain; Departamento de Medicina, Facultad de Medicina, Hospital Universitario Virgen del Rocío, Universidad de Sevilla, Seville, Spain; Centro de Investigación Biomédica en Red de Epidemiología y Salud Pública, Madrid, Spain; School of Veterinary Medicine and Science, University of Nottingham, Sutton Bonington, Loughborough, UK

**Keywords:** candidiasis, kinkajous, new lineage, *Potos flavus*, procyonid, *Pneumocystis*

## Abstract

An 11-mo-old, intact male captive kinkajou (*Potos flavus*) was submitted for postmortem investigation because of emaciation and hindlimb overgrooming. Histologically, alveolar airspaces were filled with fungal structures that were morphologically and histochemically consistent with *Pneumocystis* spp. PCR of pulmonary tissue was negative for canine distemper virus and positive for *Pneumocystis* spp. Molecular testing yielded amplification of the *Pneumocystis* spp. mitochondrial large-subunit rRNA (mtLSU rRNA, 510 bp) and the small-subunit rRNA (mtSSU rRNA, 565 bp). Phylogenetic analysis suggested a potentially novel *Pneumocystis* lineage associated with *P. flavus*. Additional nuclear loci are required to confirm its taxonomic status. Gastric and colonic histologic findings included concurrent candidiasis and colonic nematodosis. An underlying immunosuppressive disease was suspected. Further investigation is required to clarify the role of kinkajous in the ecology of fungal pathogens and the causes of immunosuppression in this species, particularly in the context of human–wildlife interactions. Enhanced surveillance and interdisciplinary collaboration are essential to evaluate potential zoonotic risks and inform conservation and public health strategies.

Kinkajous (*Potos flavus*)—medium-sized, predominantly frugivorous, quadrupedal mammals in the family *Procyonidae*—are related to raccoons, coatis, red pandas, ringtails, and olingos.^5,16^ Singular anatomic features encompass a prehensile tail and a long, narrow tongue adapted to the consumption of honey. Native habitats include the canopies of neotropical primary rainforests throughout Central America and northern South America.^5,16^ Kinkajous are often found in zoologic collections and as privately owned pets.^18^

The scientific literature describing pathologic conditions in kinkajous is scarce and includes: natural and vaccine-induced morbilliviral infection, hypertrophic cardiomyopathy with heart failure, nasal adenocarcinoma, mandibular cystic fibrous osteodystrophy of nutritional origin, and infection by the zoonotic roundworm *Baylisascaris procyonis*.^4,6,9,10,12^ Zoonotic cutaneous blastomycosis and hand cellulitis with abscess formation associated with kinkajou bites have been reported in humans.^7,8^ Additionally, a novel rabies virus variant was reported in a kinkajou from Brazil in 2020, raising concerns about the capacity of kinkajous to serve as a reservoir or spillover host for lyssaviruses.^3^ Overall, the documentation of kinkajou lesions remains highly fragmented, often limited to isolated case reports and anecdotal observations. We provide histologic and molecular descriptions of a proposed novel *Pneumocystis* lineage in a kinkajou with *Candida* spp. coinfection.

An 11-mo-old, 475-g, intact male kinkajou was submitted to the Veterinary Pathology Centre at the University of Surrey (**VPC-US**; Guildford, Surrey, UK) in March 2024 for postmortem examination. Clinical history included recent weight loss and overgrooming of hindlimbs and feet. The kinkajou had been added to the zoologic collection 3 mo before death and was housed with other species, including ferrets. Implementation of recent dietary changes were noted in the submission history.

Premortem zinc sulphate fecal flotation (Idexx) was negative for parasite oocysts and cysts. Antemortem bloodwork included manual interpretation of a blood smear as well as a biochemistry panel (Idexx; **Suppl. Table 1**). Blood smear evaluation did not include an estimate of the total WBC count, but did include a differential count, with left-shift neutrophilia (80% segmented; 9% bands) with toxic changes (Döhle bodies). No morphologic abnormalities were noted within the RBCs, lymphocytes (9%), monocytes (1%), or eosinophils (1%). The kinkajou was hyperglycemic (16.4 mmol/L; RI: 3.5–10.5 mmol/L) and had mildly elevated creatine kinase activity (1,290 U/L; RI: 0–673 U/L). All other biochemical analytes were within RIs. Overall, interpretation of hematology was limited by the lack of a total WBC count estimate. Biochemistry results were interpreted using published procyonid RIs.^12,13^

Grossly, the animal was in moderately poor body condition based on scant subcutaneous and visceral adipose stores as well as decreased axial and hypaxial muscle mass. The lung lobes had poorly delimited, tan-to-pale-yellow, slightly firm, raised areas, prominently distributed within the dorsal areas of the caudal lung lobes. The stomach was markedly distended by gas and had numerous, well-demarcated, dark-red, and slightly depressed ulcers covered by strings of fibrin. Samples of brain, heart, trachea, lungs, liver, kidneys, urinary bladder, testes, spleen, stomach, small intestine, large intestine, pancreas, thyroid and parathyroid glands, skeletal muscle, and sciatic nerve were placed in 10% neutral-buffered formalin. Samples of kidneys, liver, brain, lungs (including charcoal swabs of the affected areas for potential molecular or microbiologic analysis), spleen, blood, and small intestine were stored at −20°C. Selected formalin-fixed tissues (lung, kidney, liver, stomach, small and large intestine, heart, brain [cerebral cortex, pons, cerebellum, thalamus, hippocampus]) were processed routinely, sectioned, and stained with H&E for histologic evaluation.

Histologically, the alveolar septa were multifocally expanded by fibrosis and moderate numbers of histiocytes, occasional multinucleate giant cells, lymphocytes, and plasma cells. Lymphoplasmacytic perivascular infiltration was also often observed. The alveolar airspaces were often filled with proteinaceous fluid and fibrin, moderate numbers of foamy macrophages, scant neutrophils, and myriad, round-to-oval, 5–10-µm, extracellular and intracellular fungal organisms with a thin eosinophilic outline and a central slightly basophilic core (**Fig. 1A**). Histochemically, the fungi stained positively with both periodic acid–Shiff and Gomori methenamine silver stains, highlighting the fungal cell walls (**Fig. 1B, 1C**). The gastric mucosal ulcers were lined by occasional 3–6-µm wide, pale-staining, thin-walled yeast, and frequent slender, 3–4-µm wide, parallel-walled, septate pseudo-hyphae with occasional constriction points, morphologically compatible with *Candida* spp. The adjacent gastric mucosa had glandular atrophy and attenuation of the surface epithelium. The colonic mucosa was expanded by a mild-to-moderate number of lymphocytes, plasma cells, and eosinophils, and was occasionally ulcerated with numerous intralesional fungal elements with the same morphology as those observed in the stomach. Deep within the mucosa, and occasionally branching, were intralesional true hyphae (**Fig. 1D**). Moderate numbers of nematode parasites were located within colonic crypts and lumen. The nematodes had a thin cuticle, platymyarian musculature, rhabditiform esophagus, single uterus, and no apparent alae (consistent with strongyles).

**Figure 1.**
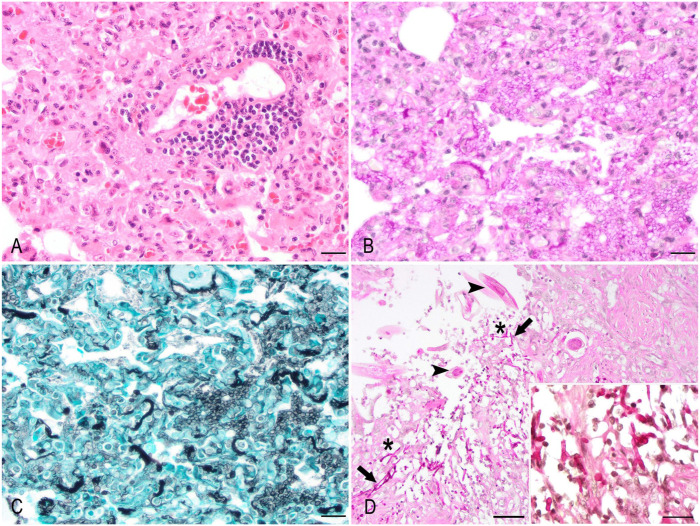
*Pneumocystis* pneumonia and colonic candidiasis in a kinkajou (*Potos flavus*). **A.** Pulmonary septa expanded by lymphocytes and plasma cells, prominent within perivascular regions, and macrophages and multinucleate giant cells. Alveoli are filled with edema, fibrin, foamy macrophages, and numerous, round-to-oval, 5–10-µm, extracellular and intramacrophagic fungal elements with a faintly eosinophilic outline and a frequent amphophilic to faintly eosinophilic granular core. H&E. Bar = 20 µm. **B.** The walls of fungal cysts in the lung, highlighted in magenta, occasionally upholster the alveolar epithelium. PAS. Bar = 20 µm. **C.** The 5–10-µm fungal cysts are round-to-crescentic, with cell walls outlined in black, and often form clusters within alveoli. Grocott–Gomori methenamine silver. Bar = 20 µm. **D.** A colonic ulcer contains 3–6-µm yeasts (inset), slender, 3–4-µm wide, parallel-walled, with occasional constrictions (pseudo-hyphae, arrows), and occasionally branching true hyphae (asterisks), compatible with *Candida* spp. Moderate numbers of intralesional nematodes (arrowheads) in both transverse and longitudinal sections in the intestinal lumen and crypts. Bar = 50 µm. Inset: detail of intralesional fungal structures. PAS. Bar = 20 µm.

PCR testing for canine distemper virus (CDV) was performed on fresh-frozen lung tissue at Laboklin (Bad Kissingen, Germany), an ISO/IEC 17025–accredited veterinary diagnostic laboratory, using a validated, proprietary assay (Service ID 8003; qualitative real-time PCR), with appropriate positive and negative controls,^14^ and was negative. PCR testing for *Pneumocystis* spp. was performed on formalin-fixed, paraffin-embedded (FFPE) tissues. Nucleic acid extraction was performed on 2 separate 20-mg sections of FFPE lung tissue. Paraffin was manually removed with a sterile scalpel, and the tissue was transferred to 1.5-mL microcentrifuge tubes for xylene-based deparaffinization. After drying, samples were digested with proteinase K, and DNA was extracted (NucleoSpin tissue kit; Macherey-Nagel) following the manufacturer’s instructions. DNA was eluted in 100 µL of ultrapure water, and concentration and purity were assessed (NanoDrop spectrophotometer; ThermoFisher).

Mitochondrial large-subunit rRNA (***mtLSU***) and small-subunit rRNA (***mtSSU***) genes were targeted using primers LSU.f1 (5′-CTCATGTCAGCATTCTCTCTTTA-3′) and LSU.r1 (5′-AGGATATAGCTGGTTTTCTGCGA-3′) for *mtLSU*, and rns.f8 (5′-GCGCTTGACGAGTAGTTAGT-3′) and rns.r13 (5′-AGATACCCTTGTAGTTTATGCTG-3′) for *mtSSU*, following published methodologies.^17^ PCR products were purified by size-exclusion ultrafiltration (Sephacryl S-400 HR; Sigma-Aldrich). Sequencing was performed (BigDye Terminator v.3.1; Applied Biosystems), purified by EDTA–ethanol precipitation, resuspended (Hi-Di formamide; ThermoFisher), and run on a genetic analyzer (AB3500; Applied Biosystems). Sequence quality was checked (SeqScanner 2; ThermoFisher) and analyzed in MEGA v.11.0.13. Finally, *mtLSU* and *mtSSU* rRNA DNA sequences were aligned using MEGA 11 software with MUSCLE (Multiple Sequence Comparison by Log-Expectation) conditions, and the maximum-likelihood phylogeny was calculated using the HKY+G model according to the Bayesian information criterion (BIC) for the *mtLSU* gene and the T92+G model for the *mtSSU* gene (ST2A, ST2B).

Lung tissue samples tested positive for *Pneumocystis mtLSU* and *mtSSU* rRNA genes by PCR, yielding identical sequences for each gene based on direct Sanger sequencing, with clean, non-overlapping peaks in chromatograms with no evidence of mixed infection (100% identity; **Suppl. Fig. 1**; submitted to GenBank: PV037717.1 for *mtSSU* RNA [S3A, S3B] gene and PV037718.1 for *mtLSU* [S3C, S3D] gene). Phylogenetic analysis identified *P. canis* as the closest relative to the detected *Pneumocystis* spp., with strong bootstrap support (99) for both genes (**Fig. 2**). Pairwise genetic distances between *P. canis* and the distinct *Pneumocystis* lineage associated with *Potos flavus* (Index Fungorum IF 904239) were 0.058 (*mtLSU*) and 0.027 (*mtSSU*), whereas the overall average distances among all analyzed species were 0.244 (*mtLSU*) and 0.130 (*mtSSU*; **Suppl. Tables 1, 2**).

**Figure 2.**
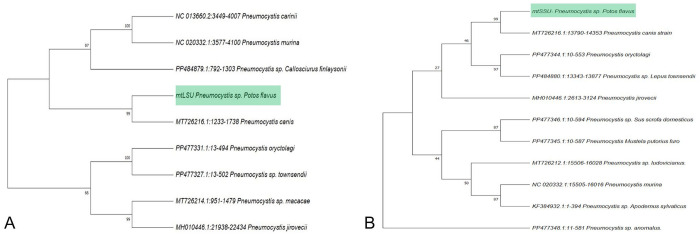
Maximum-likelihood phylogenetic trees of *Pneumocystis* in a kinkajou. **A.** Based on the *mtLSU* rRNA gene. **B.** Based on the *mtSSU* rRNA gene.

FFPE lung tissue and histologic slides from our case are archived at the VPC-US. Given that only mitochondrial loci were obtained, we conservatively refer to this organism as *Pneumocystis* sp. (*Potos flavus* lineage). Further support for its designation as a distinct species would require sequencing and phylogenetic analysis of nuclear loci, which were not available for this sample and represent a major limitation for definitive species identification.^11^

*Pneumocystis* is a genus of highly host-specific, ubiquitous fungi that infect mammals opportunistically. The 7 formally named species include: *P. jirovecii* (human), *P. murina* (mice), *P. carinii* (swine and rats), *P. oryctolagi* (rabbits), *P. canis* (dogs), *P. wakefieldiae* (rats), and *P.* ‘macacae’ (macaques).^2^ Presumably, the life cycle alternates between 2 major stages including the cyst or ascus (5–8 µm), which are thick-walled cysts containing intracystic bodies, and the metabolically active trophic forms (2–8 µm), which attach to type I pneumocytes.^2^ Although pathologic effects of *Pneumocystis* spp. are most prevalent in young and immunosuppressed individuals, subclinical infection in presumed immunocompetent individuals has been reported.^17^ Animals, including humans, may acquire *Pneumocystis* spp. through several routes: horizontal airborne transmission from infected or subclinical hosts, vertical transmission through the placenta, which has been postulated in rabbits and humans, or perinatally through contact with colonized or infected parents during the neonatal period.^1^

Hematologically, our case had left-shifted neutrophils with toxic changes, suggestive of rapid neutropoiesis in response to the pneumonia and mycotic gastrocolitis. Hyperglycemia was likely part of an acute stress response, which has been documented in both companion and wildlife animals.^15^

Lymphoplasmacytic-to-granulomatous interstitial pneumonia with fibrosis is typically described in pneumocystosis in animals.^17^ The granulomatous and lymphoplasmacytic interstitial pneumonia in our case was prominent within caudodorsal areas of the lungs, with abundant intra-alveolar pneumocystis organisms, aligning with published reports.^17^

The multifocal perivascular accumulation of lymphocytes and plasma cells within the lung tissue in our case raised additional concern for an underlying primary infectious condition that could have predisposed this animal to opportunistic infections. Reported infectious comorbidities in animals infected with *Pneumocystis* spp. include CDV, canine parvovirus, feline panleukopenia virus, feline leukemia virus, porcine circovirus 2, and porcine reproductive and respiratory virus, among others.^17^ Vaccine-induced and naturally occurring CDV have been reported in kinkajous.^9^ Interestingly, our case was housed in a collection with ferrets that died following a feline panleukopenia outbreak. Testing for potential parvoviral infection was not performed in our case as neither gross nor histologic findings (i.e., enteritis and/or Peyer patch necrosis) were consistent with such infection.

Additionally, ulcerative gastritis and colitis with intralesional fungal elements morphologically compatible with *Candida* spp. were observed in our case, with concomitant nematodosis in the colonic lesions. Coinfection of *Pneumocystis* spp. and *Candida albicans* has been described in a wide range of animal species with compromised immune status.^16^ Kinkajous have been documented to harbor gastrointestinal nematodes (e.g., *Ancylostoma* spp., *Strongylus* spp.), resulting in damage to the intestinal mucosa and chronic enteritis, potentially creating idoneous conditions for opportunistic organisms.^12,20^ In addition, recent acquisition and dietary change were reported, potentially resulting in increased stress. Gastric ulcers related to husbandry stress and abrupt dietary change have been described in other captive exotic species.^19^ We suspect opportunistic colonization of the damaged gastric mucosa by *Candida* spp. in our case.

## Supplemental Material

sj-pdf-1-vdi-10.1177_10406387251413289 – Supplemental material for Pulmonary pneumocystosis in a captive kinkajou with molecular evidence of a novel Pneumocystis lineage Supplemental material, sj-pdf-1-vdi-10.1177_10406387251413289 for Pulmonary pneumocystosis in a captive kinkajou with molecular evidence of a novel Pneumocystis lineage by Pablo Díaz-Santana, Alejandro Suárez-Bonnet, Javier Déniz-Marrero, Francisco J. Salguero, Bernat Martí-Garcia, Vincente Friaza, Enrique J. Calderón and Sai Fingerhood in Journal of Veterinary Diagnostic Investigation
