# Supplementary material for: Pulmonary pneumocystosis in a captive kinkajou with molecular evidence of a novel Pneumocystis lineage
Source: J Vet Diagn Invest. 2026 Jan 22:10406387251413289. Online ahead of print. doi: 10.1177/10406387251413289 (PMC12830342; doi:10.1177/10406387251413289)
Supplement: sj-pdf-1-vdi-10.1177_10406387251413289 – Supplemental material for Pulmonary pneumocystosis in a captive kinkajou with molecular evidence of a novel Pneumocystis lineage [file sj-pdf-1-vdi-10.1177_10406387251413289.pdf]

JVDI: Supplemental material

Díaz-Santana P, et al. Pulmonary pneumocystosis in a captive kinkajou with molecular evidence of a novel *Pneumocystis* lineage

Supplemental Table 1A. Hematologic values in a kinkajou (*Potos flavus*) with pulmonary pneumocystosis.

Parameter	Result	RI*
Neutrophils, %	80.0	—
Band neutrophils, %	9.0	—
Lymphocytes, %	9.0	—
Monocytes, %	1.0	—
Eosinophils, %	1.0	—
Basophils, %	0.0	—

* No laboratory-specific RIs were available for *P. flavus*. Differential percentages are presented for descriptive purposes only and should not be interpreted without a total WBC count. Species-specific RIs for *P. flavus* are from Ratliff C, Heatley JJ. Procyonids. In: Heatley JJ, Russell KE, eds. Exotic Animal Laboratory Diagnosis. Wiley, 2020:98–112.

Supplemental Table 1B. Serum biochemistry profile in a kinkajou with pulmonary pneumocystosis.

Analyte	Result	RI*
Glucose, mmol/L	16.4	2.8–10.9
Urea, mmol/ L	7.7	1.1–11.8
Creatinine, µmol/L	27	18–88
Total protein, g/L	80	61–91
Albumin, g/L	34	29–46
Globulin, g/L	46	27–54
Albumin:globulin	0.7	~0.5–1.5
ALT, U/L	85	18–107
AST, U/L	258	90–316
ALP, U/L	15.0	14–124
GLDH, U/L	5.4	Not available
CK, U/L	1,290	0–673 U/L

ALT = alanine aminotransferase; AST = aspartate aminotransferase; ALP = alkaline phosphatase; GLDH = glutamate dehydrogenase; CK = creatine kinase.

* Species-specific RIs for the kinkajou (*P. flavus*) are from Ratliff C, Heatley JJ. Procyonids. In: Heatley JJ, Russell KE, eds. Exotic Animal Laboratory Diagnosis. Wiley, 2020:98–112.

Supplemental Table 2A. Pairwise genetic distance among *Pneumocystis* spp. from different animals at *mtLSU* RNA sequences.

	No. of sequences used per organism							
Organism	1	2	3	4	5	6	7	8
mtLSU *Pneumocystis* sp. *Potos flavus*								
mtLSU *Pneumocystis canis*	0.0577							
mtLSU *Pneumocystis* sp. *Callosciurus finlaysonii*	0.2436	0.2617						
mtLSU *Pneumocystis oryctolagi*	0.2112	0.2420	0.2595					
mtLSU *Pneumocystis* sp. *townsendii*	0.2196	0.2244	0.2817	0.1020				
mtLSU *Pneumocystis* sp. *macacae*	0.2181	0.2090	0.2325	0.2620	0.2592			
mtLSU *Pneumocystis jirovecii*	0.2392	0.2405	0.2435	0.2598	0.2628	0.1368		
mtLSU *Pneumocystis carinii*	0.2951	0.2939	0.2674	0.3211	0.3258	0.2763	0.2877	
mtLSU *Pneumocystis murina*	0.2683	0.2920	0.2345	0.2954	0.3009	0.2822	0.2533	0.1257

Estimates of Evolutionary Divergence between Sequences. The number of base substitutions per site from between sequences is shown. Analyses were conducted using the maximum composite likelihood model [1]. This analysis involved 9 nucleotide sequences. Codon positions included were 1st+2nd+3rd+Noncoding. All ambiguous positions were removed for each sequence pair (pairwise deletion option); 605 positions in the final dataset. Evolutionary analyses were conducted in MEGA11.

Supplemental Table 2B. Pairwise genetic distance among *Pneumocystis* spp. from different animals at *mtSSU* sequences.

	No. of sequences									
Organism	1	2	3	4	5	6	7	8	9	10
mtSSU *Pneumocystis* sp. *Potos flavus*										
mtSSU *Pneumocystis murina*	0.1451									
mtSSU *Pneumocystis canis* strain	0.0272	0.1451								
mtSSU *Pneumocystis* sp. *Sus scrofa domesticus*	0.1383	0.1115	0.1315							
mtSSU *Pneumocystis* sp. *Mustela*	0.1367	0.0983	0.1366	0.0842						
mtSSU *Pneumocystis oryctolagi*	0.1878	0.1667	0.1828	0.1761	0.1802					
mtSSU *Pneumocystis* sp. *anomalus*	0.1239	0.1397	0.1194	0.1342	0.1261	0.1735				
mtSSU *Pneumocystis* sp. *ludovicianus*	0.1158	0.1005	0.1134	0.0897	0.0808	0.1668	0.1104			
mtSSU *Pneumocystis* sp. *Lepus townsendii*	0.1424	0.1404	0.1398	0.1549	0.1472	0.0790	0.1573	0.1403		
mtSSU *Pneumocystis jirovecii*	0.1664	0.1628	0.1637	0.1402	0.1493	0.1549	0.1521	0.1503	0.1475	
mtSSU *Pneumocystis* sp. *Apodemus sylvaticus*	0.1124	0.0623	0.1123	0.0776	0.0897	0.1359	0.0983	0.0797	0.1203	0.1528

Estimates of Evolutionary Divergence between Sequences. The number of base substitutions per site from between sequences is shown. Analyses were conducted using the maximum composite likelihood model. This analysis involved 11 nucleotide sequences. Codon positions included were 1st+2nd+3rd+Noncoding. All ambiguous positions were removed for each sequence pair (pairwise deletion option); 606 positions in the final dataset. Evolutionary analyses were conducted in MEGA11.

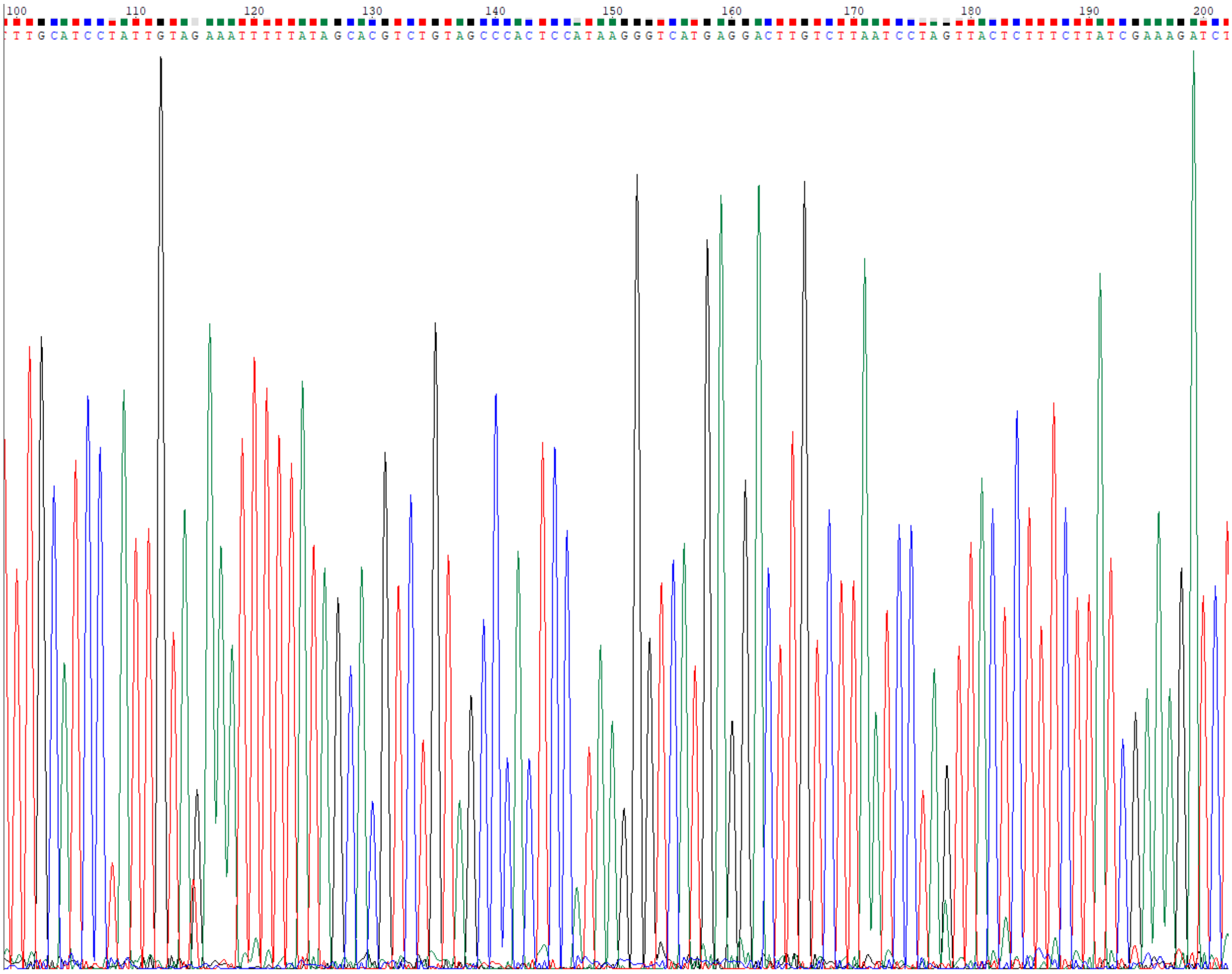

Supplemental Figure 1A. Representative Sanger sequencing chromatogram of the mitochondrial small-subunit rRNA (*mtSSU*) gene (forward strand, sample mtSSU-F1-1). Clear, distinct peaks indicate high-quality base calling across.

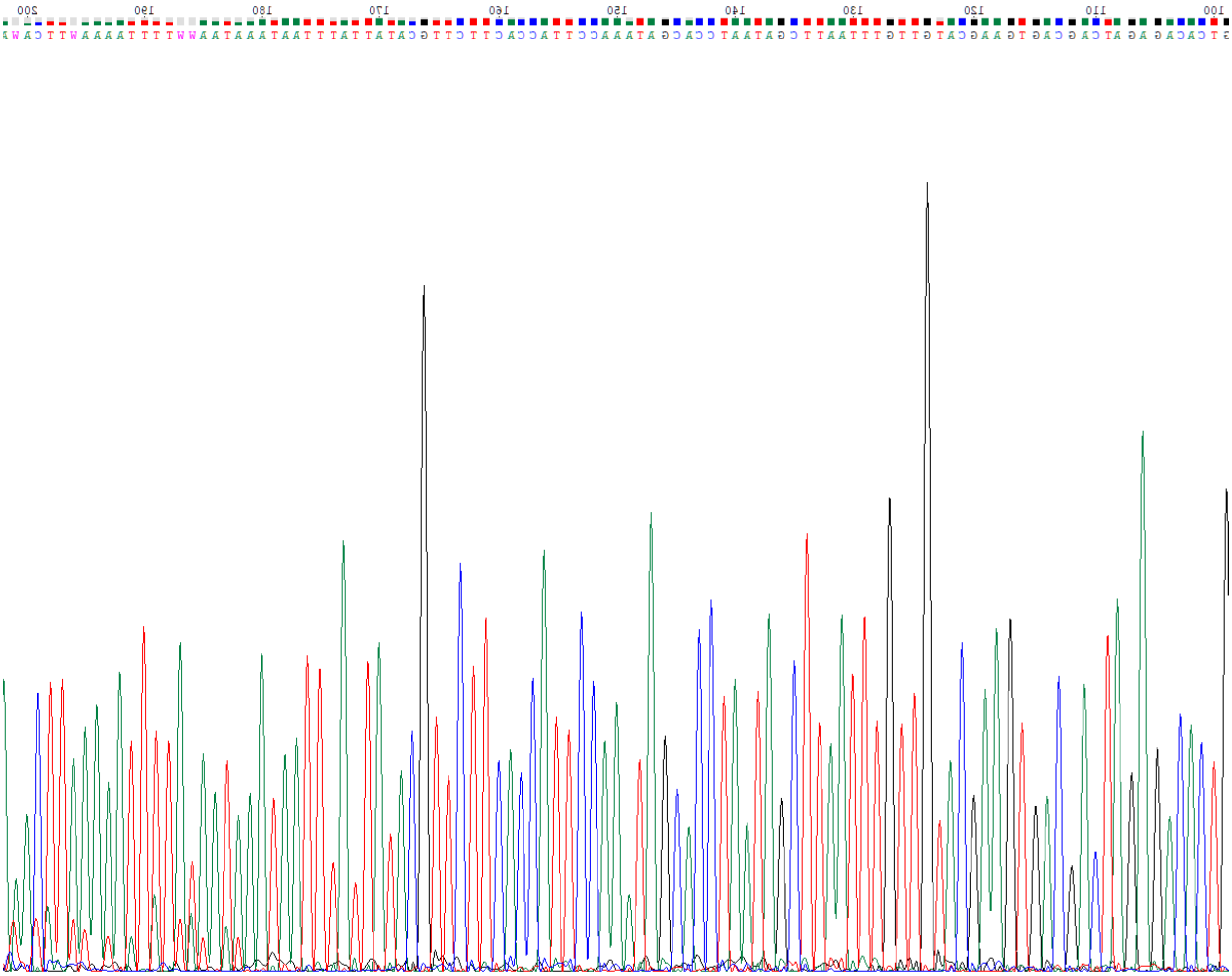

Supplemental Figure 1B. Representative Sanger sequencing chromatogram of the mitochondrial small-subunit rRNA (*mtSSU*) gene (reverse strand, sample mtSSU-R1-1). Well-defined peaks demonstrate accurate base discrimination and concordance with the forward strand sequence.

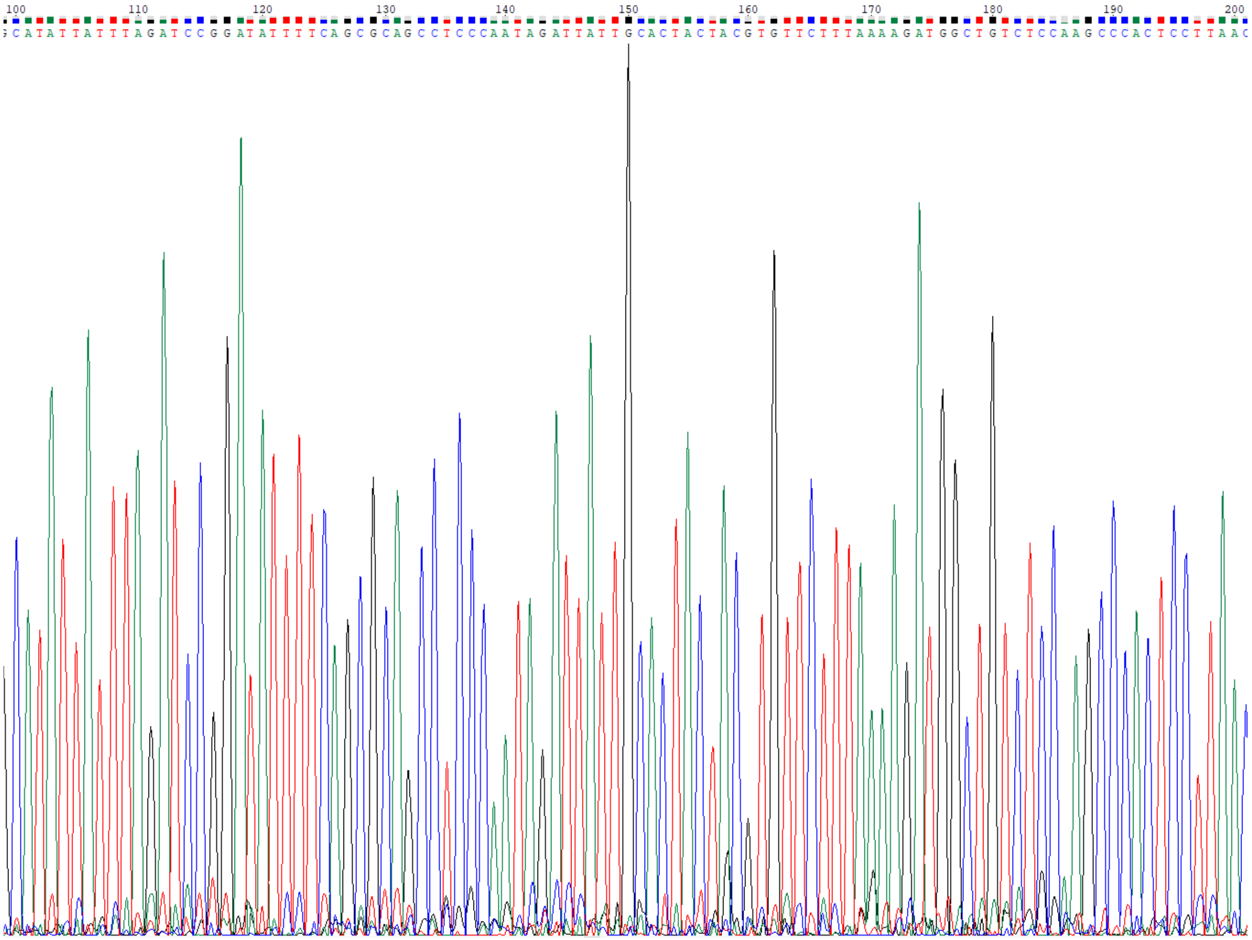

Supplemental Figure 1C. Representative Sanger sequencing chromatogram of the mitochondrial large-subunit rRNA (*mtLSU*) gene (forward strand, sample mtLSU-F1-1). Clear, evenly spaced peaks indicate high-quality read and reliable sequence determination.

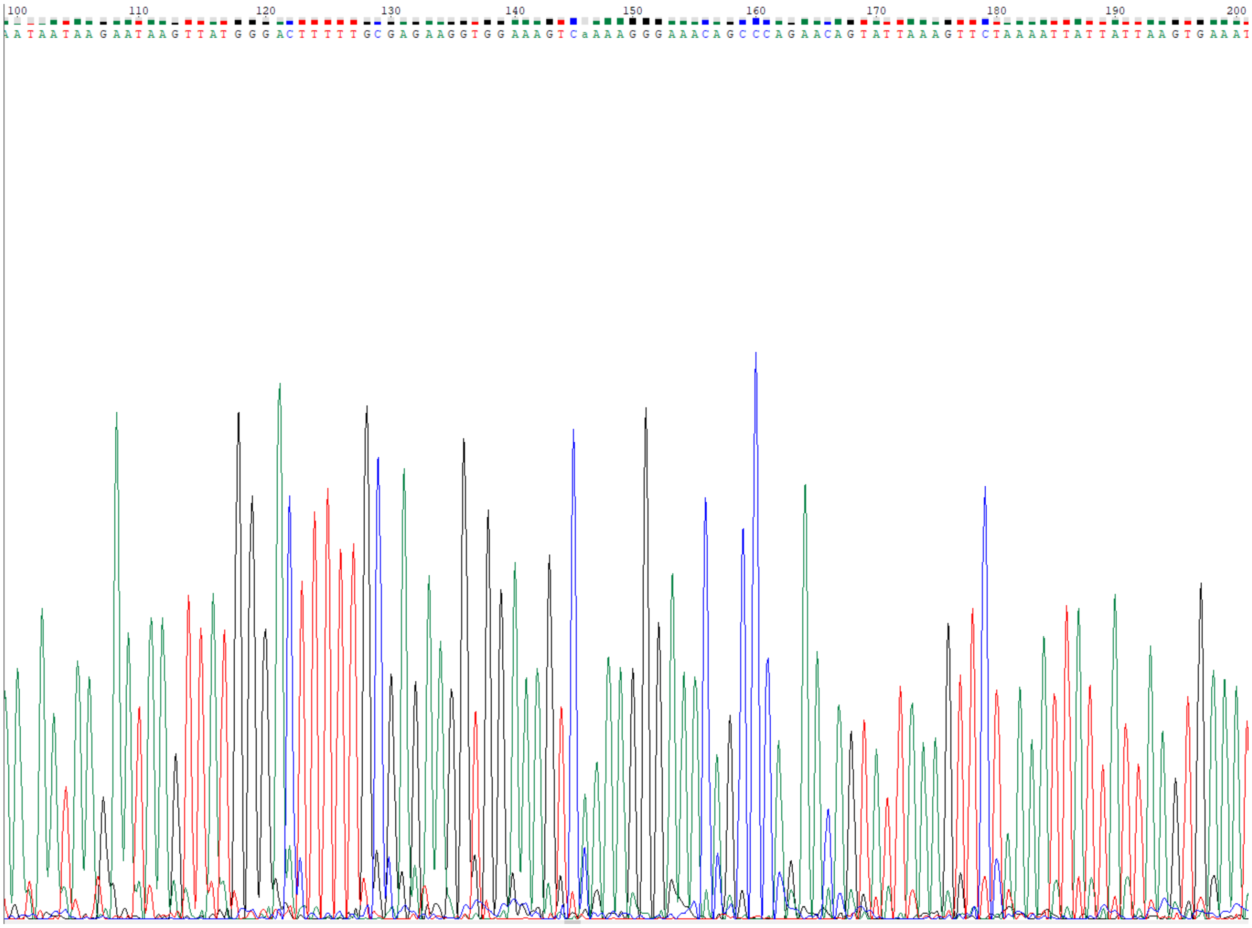

Supplemental Figure 1D. Representative Sanger sequencing chromatogram of the mitochondrial large-subunit rRNA (*mtLSU*) gene (reverse strand, sample mtLSU-R1-1). Distinct peak morphology confirms high-quality reverse read alignment with the forward strand.
